# Potential of Biological Processes to Eliminate Antibiotics in Livestock Manure: An Overview

**DOI:** 10.3390/ani4020146

**Published:** 2014-04-04

**Authors:** Daniel I. Massé, Noori M. Cata Saady, Yan Gilbert

**Affiliations:** Dairy and Swine Research and Development Centre, Agriculture and Agri-Food Canada, Sherbrooke, Quebec, J1M 0C8, Canada; E-Mail: Noori.saady@agr.gc.ca (N.M.C.S.); gilbertyan@hotmail.com (Y.G.)

**Keywords:** antibiotics, livestock, manure, degradation, fate, anaerobic digestion

## Abstract

**Simple Summary:**

Beside their use to treat infections, antibiotics are used excessively as growth promoting factors in livestock industry. Animals discharge in their feces and urine between 70%–90% of the antibiotic administrated unchanged or in active metabolites. Because livestock manure is re-applied to land as a fertilizer, concerns are growing over spread of antibiotics in water and soil. Development of antibiotic resistant bacteria is a major risk. This paper reviewed the potential of anaerobic digestion to degrade antibiotics in livestock manure. Anaerobic digestion can degrade manure-laden antibiotic to various extents depending on the concentration and class of antibiotic, bioreactor operating conditions, type of feedstock and inoculum sources.

**Abstract:**

Degrading antibiotics discharged in the livestock manure in a well-controlled bioprocess contributes to a more sustainable and environment-friendly livestock breeding. Although most antibiotics remain stable during manure storage, anaerobic digestion can degrade and remove them to various extents depending on the concentration and class of antibiotic, bioreactor operating conditions, type of feedstock and inoculum sources. Generally, antibiotics are degraded during composting > anaerobic digestion > manure storage > soil. Manure matrix variation influences extraction, quantification, and degradation of antibiotics, but it has not been well investigated. Fractioning of manure-laden antibiotics into liquid and solid phases and its effects on their anaerobic degradation and the contribution of abiotic (physical and chemical) *versus* biotic degradation mechanisms need to be quantified for various manures, antibiotics types, reactor designs and temperature of operations. More research is required to determine the kinetics of antibiotics’ metabolites degradation during anaerobic digestion. Further investigations are required to assess the degradation of antibiotics during psychrophilic anaerobic digestion.

## 1. Introduction

Feeding antimicrobials (antibiotics) as growth promoter at sub-therapeutic doses to swine, cattle, poultry, and fish [1,2] is an integral part of the farm animal/fish production. Antibiotics are relatively recalcitrant to degradation. At significant concentrations, they impose bactericidal or antimicrobial effects which inhibit bacterial activity or growth. Animals excrete a significant fraction of antibiotics in feces and urine; therefore, there is substantial risk that unaltered or still active metabolites would be found in the environment. Different pathways for antibiotics introduction into the environment within an agricultural context were suggested [3]. Land application of livestock manure spreads antibiotics into environment at large scale. The excretion of wastes by grazing animals, atmospheric dispersal of feed and manure dust containing antibiotics [4] and the incidental release of products from spills or discharge are also potential pathways introducing antibiotics into the environment. Antibiotics in food products from animals and plants [5], the development and spread of antibiotic resistant bacteria [6], and the aquatic environments contamination from manure land application are concerns about agricultural antibiotic usage.

### 1.1. Antibiotic Consumption in Livestock Industry

**Figure 1:**
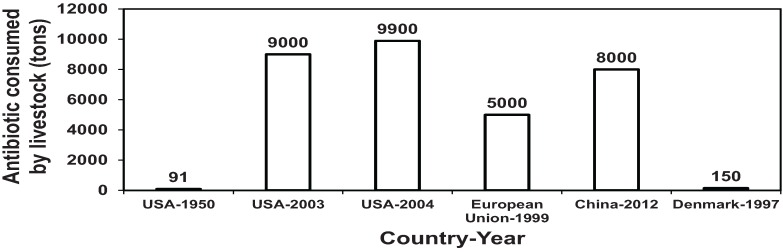
Quantities of antibiotics consumed by livestock in animal feed (Data from [7,8,9,11]).

Antibiotic consumption in livestock industry in USA, European countries and China is given in Figure 1. Notice that in the USA, for example, the quantity of antibiotics used in 2004 is 108 times that used in 1950. This is partially because the recommended levels of growth-promoting antibiotics in poultry and pig diets increased from 4 ppm for the narrow spectrum and 10 ppm for the broad-spectrum antibiotics in 1950s to 200 ppm nowadays. About 91% of livestock operations in the USA use 11.2 million kg antibiotics sold over-the-counter as growth promoters annually [6,7,8,9,10]. Antibiotics fed to animals end up in manure and eventually in the environment.

### 1.2. Types of Antibiotics Used in Livestock

Major antibiotics used in livestock: Various antibiotics classes are used to various extents and frequencies therapeutically and sub-therapeutically in livestock industry [2] including:

1. b-Lactams: *penicillins*: amoxicillin, ampicillin, benzylpenicillin, cloxacilin, dicloxacilin, flucloxacillin, methicillin, mezlocillin, nafcillin, oxacillin, piperacillin, phenoxymethylcillin.
2. Macrolides: azithromycin, clarithromycin, clindamycin, *erythromycin*, roxithromycin, spiramycin, *tylosin*, vancomycin.
3. Sulphonamides: *sulphadimidine*, *sulphamethoxazole*.
4. *Trimethoprim*.
5. Fluorochinolones: ciprofloxacin, ofloxacin.
6. *Tetracyclines*: *chlortetracycline, doxycycline, oxytetracycline, tetracycline*.
7. Polyether antibiotic: *monensin*

The antibiotics in Italic font are the major antibiotics usually used in swine and cattle while other antibiotics are less frequent. Pan *et al.* [12] reported detection frequencies of 85%–97% (tetracyclines), 52% (sulphonamides), and 5% (macrolide) in 126 swine manure samples collected from 21 animal feeding operation in Shandong-China. Similar results were reported in China (Chen *et al.* [11] and Japan [13]). Tetracyclines (especially oxytetracycline (OTC) and chlortetracycline (CTC)) occur worldwide in lagoon samples or manures from livestock husbandry [14,15,16,17,18].

### 1.3. Excretion of Antibiotics in Livestock Manure

Animals excrete significant proportion of antibiotics (17%–90% for livestock) [7,19,20,21] directly into urine and feces, unchanged or as active metabolites (epimers or isomers) of the parent species [22]. Table 1 gives the percentage of antibiotics excreted by animals with their metabolic status (changed or unchanged from their administrated form). Some metabolites are more potent than their parent compounds, while others such as acetic conjugates of sulphonamides can revert back to their parent compounds during manure storage [23].

**Table 1:** Level of excretion of antibiotics from animals.

Antibiotic	Source of manure	Excretion level (%)	Status	Reference
Chlortetracycline	Steers feces	75	Not reported	[24]
Tetracycline	Animal feces	25	Not reported	[25]
Tylosin	Urine	50–60	Unchanged	[25]
Oxytetracycline	Castrate sheeps	21	Unchanged	[26]
Chlortetracycline	Young bulls	17–75	Unchanged	[26]
Tylosin	Pigs	40	Unaltered or as potent metabolites	[27]
Monensin	Beef cattle feces	40%	Unchanged	[28]
Virginiamycin	Piggeries liquid manure	20	After several days of storage	[29]
Oxytetracycline	Calves manure (feces, urine, and bedding)	23	Not reported	[30]

### 1.4. Concentration of Antibiotics in Livestock Manure

Typically, antibiotic concentrations in manure are between 1 to 10 mg·kg^−1^ or L^−1^ but may reach levels ≥ 200 mg·kg^−1^ or L^−1^ [20]. Concentrations of 100’s mg·kg^−1^ or mg·L^−1^ of veterinary antibiotics have been found in animal excreta in China [11,31,32]. It is not clear whether the high variation in the detected concentrations and the antibiotics excretion by animals were due to individual differences regarding antibiotics metabolism or to an inadequate extraction and quantification methods used during these studies. The concentration of the most commonly used antibiotics have been reported to be as high as 216 mg·L^−1^ of swine, beef, and poultry/turkey manures [21].

Several studies have confirmed that antibiotics used in animal production are present in fresh manure, manure storage tanks, soil, surface and underground water [33,34]. Jacobsen and Halling-Sørensen [18] detected tetracycline and sulphonamides in swine manure, but no tylosin was detected because of poor recoveries of tylosin from manure. De Liguoro *et al.* [35] found 0.11 mg·kg^−1^ of tylosin and 10 mg·kg^−1^ of OTC in fresh calf manure, but found negligible concentrations of these compounds in soil and water. Dolliver and Gupta [36] found that 1.2% to 1.8% chlortetracycline, monensin and tylosin were lost from manure stockpile by runoff water. Campagnolo *et al.* [37] found significant quantity of macrolides, sulphonamides and fluoroquinolones in the nearby surface water.

While the measured concentrations shown in Table 2 and elsewhere assess for the presence of antibiotics in various environmental samples and collectively provided strong evidences of their widespread in manure, each study reports its own quantification technique with particular recovery efficiencies, sensibility and reliability.

**Table 2:** Concentration of some antibiotics in manures.

Antibiotic	Matrix	Concentration	Reference
Oxytetracycline	Manure	136 mg·L^−1^	[14]
Chlortetracycline		46 mg·L^−1^	
Tetracycline	Swine manure	98 mg·L^−1^	[11]
Oxytetracycline		354 mg·L^−1^	
Chlortetracycline		139 mg·L^−1^	
Doxycycline		37 mg·L^−1^	
Sulfadiazine		7.1 mg·L^−1^	
Tetracycline	Swine manure	30 mg·kg^−1^ DM	[18]
Sulphonamides		2 mg·kg^−1^ DM	
Tylosin	Fresh calf manure	0.11 mg·kg^−1^	[35]
Oxytetracycline		10 mg·kg^−1^	
Chlortetracycline,	Beef manure stockpile	6.6 mg·kg^−1^	[36]
Monensin		120 mg·kg^−1^	
Tylosin		8.1 mg·kg^−1^	
Oxytetracycline	Cow manure	0.5–200 mg·L^−1^	[38]
Chlortetracycline	Swine manure	764.4 mg·L^−1^	[12]
Chlortetracycline	Swine manure storage lagoon	1 mg·L^−1^	[37]
Oxytetracycline		0.41 mg·L^−1^	

There is no standardized and reliable method for antibiotics quantification in complex matrices, such as soil and biological sludge, making inter- and even intra-study comparisons difficult. Most studies report results without sufficiently describing the condition of the manure handling and management before sampling. Partitioning of antibiotics into the liquid and solid phases affects the results. For example, sampling a leaching manure pile would indicate the solid phase fraction of the antibiotics rather than the total concentration.

Most of the antibiotic residues in manure form complexes with soluble organics and remain stable during manure storage. When manure is applied to agriculture fields, a fraction of the antibiotics becomes mobile with the flow of water in the soil and contaminate the surrounding environment including surface and groundwater. The extent of fractioning of an antibiotic between solid and aqueous phases, and hence its mobility, depends on the properties of the antibiotic, soil, and the hydrological effects. More research is required to understand kinetics of biodegradation and potencies of degraded products of various antibiotics in different environments (soils, manures and waste water).

### 1.5. Environmental Transport of Antibiotics from Livestock Manure

Antibiotics behavior and transport in the environment are related to their physicochemical properties [33]. Numerous antibiotics comprise a non-polar core associated with polar functional moieties; many antibiotics are amphiphilic or amphoteric and ionized. However, physicochemical properties vary widely among compounds from the various structural classes and antibiotics of the same class do not necessarily exhibit identical behaviors. Adsorption of antibiotics to the organic and mineral exchange sites in soil is mostly due to charge transfer and ion interaction and not only to hydrophobic partitioning [34].

Wu *et al.* [39] demonstrated that ciprofloxacin, tetracycline, doxycycline, and clindamycin were strongly sorbed on aerobically digested biosolids, while sulfamethazine and sulfamethoxazole were only weakly sorbed to particles. Davis *et al.* [40] investigated the transport of seven different antibiotics used in animal production during a simulated rainfall event and determined their association with the sediment or the aqueous phase. They reported that the percentage of partitioning of the antibiotics into (aqueous, solid) phases for sulfathiazole (77,23), sulfamethazine (95,5), monensin (91,9), erythromycin (26,74), and tylosin (23,77). Therefore, sulfathiazole, sulfamethazine, and monensin mostly associated with aqueous phase while tylosin and erythromycin associated with the solid phase. The tendency of some antibiotics to adsorb on particles reduces their bioavailability [39,41] and results in low degradation rates. Chen *et al.* [11] found tetracycline, oxytetracycline, chlortetracycline, and doxycycline (0.1–205 μg·kg^−1^) in manure-amended soils near swine farms. Dolliver *et al.* [5] found that corn, lettuce, and potato took sulfamethazine from a manure-amended soil and accumulated 0.1 mg to 1.2 mg sulfamethazine kg^−1^ of dry plant tissue after 45 days of growth; although the accumulated concentration is relatively low it might still pose a health concern. Therefore, surface waters, agricultural soils, and groundwaters may become reservoirs to antibiotics because of the current manure management practices [42,43]. Many antibiotics have short half-lives (days to weeks) [41,44], but at high concentration some persist for months to years within agricultural-related matrices [27,45]. For example, manure storage does not affect tetracyclines and sulfadiazine [11]. Lamshöft *et al*. [46] observed that metabolites of sulfadiazine have been reversibly converted to sulfadiazine; therefore, they suggested that frequent fertilization of soil by manure contaminated with sulfadiazine and its metabolites may cause them to accumulate in soil and results in environmental contamination. The physicochemical properties, the structure of antibiotics and their degradation by-products determine whether they degrade during biological treatment [47], some compounds and their metabolites may persist for days to months [11].

Evaluating the data available on degradation and fate of antibiotics during anaerobic digestion of livestock manure is essential to the development of this technology as an integral part of the strategy to control the spread of antibiotic resistant bacteria. This paper summarizes what is currently known about behavior, of the major antibiotic used in livestock therapeutically or as growth promoters, during anaerobic digestion, their metabolic by-products, and fractioning into aqueous and solid fractions. There is little information regarding the effect and fate (removal) of antibiotics during the anaerobic digestion of manure [30,48].

## 2. Persistence and Biodegradation of Antibiotics during Biological Processes of Manure Treatment

### 2.1. Persistence of Antibiotics in Manure

A summary of the reported half-life (*t*_1/2_) of some antibiotics during manure storage or in soil environment is given in Table 3. Generally, a wide range of half-lives has been reported in the scientific literature regarding antibiotic degradation from different environmental conditions.

**Table 3:** Half life of antibiotics under storage and natural environment conditions.

Antibiotic	Medium matrix	Half-life (days unlessindicated otherwise)	Reference
Tetracycline	Biosolids storage	37 to >77	[49]
Tetracycline	Stored feedlot manure	17.2	[50]
Chlortetracycline	Composted manure	3	[51]
Chlortetracycline	Dairy manure	6.8	[50]
Chlortetracycline	Stored feedlot manure	13.5	[50]
Oxytetracycline	Stockpiled fresh manure (low-intensity composting)	21	[35]
Oxytetracycline	Dairy manure	17.7	[50]
Oxytetracycline	Stored feedlot manure	31.1	[50]
Oxytetracycline	Horse manure	8.4	[50]
Tylosin	Aerobic soil-manure slurry	3.3–8.1	[41]
Olaquindox	Aerobic soil-manure slurry	5.8–8.8	[41]
Metronidazole	Aerobic soil-manure slurry	13.1–26.9	[41]
Erythromycin	Storage of pig manure	41	[52]
Erythromycin	Biosolids storage	7.0-17	[49]
Roxithromycin	Storage of pig manure	130	[52]
Salinomycin	Storage of pig manure	6	[52]
Doxycycline	Biosolids storage	53 to >77	[49]
Clindamycin	Biosolids storage	1.0–1.6	[49]
Clarithromycin	Biosolids storage	1.1–1.9	[49]

Storteboom *et al.* [50] found that OTC persists in dairy manure (*t*_1/2_ = 17.7 d) longer than in horse manure (*t*_1/2_ = 8.4 d). However, matrix differences could have influenced antibiotic recoveries during extraction and quantification methods used and thus biased half-lives.

In addition, different types of biological processes affect antibiotic persistence differently; for example, chlortetracycline half-life has been found to increase in the order: composting > manure storage > soil. Half-lives for the primary degradation in aerobic soil-manure slurries ranging from 3.3 to 8.1 days for tylosin, 5.8 to 8.8 days for olaquindox, and 13.1 to 26.9 days for metronidazole were observed [41]. Schlüsener *et al.* [53] indicated that erythromycin, roxithromycin and salinomycin, tetracycline, doxycycline, clindamycin, and clarithromycin were more persistent under anaerobic conditions than aerobic condition with a longer *t*_1/2_ by a factor of 1.5 to 2, suggesting that aerobic degradation might be a more important mechanism to eliminate these compounds from the environment [49]. However, the results were not sufficiently strong to support this assumption. The authors emphasized on the need to obtain more data for compounds from different classes to support this assumption and to perform further research to clarify the degradation pathways and identify the metabolites. Also, the poor antibiotic recoveries related to the extraction techniques used (31%–83% recoveries) and high matrix effects (50%–90%) can account in part for the variation in results. Table 4 gives the half-lives of major antibiotic classes in manure environment; notice that tetracyclines and quinolones are very persistent with an average half-live of around 100 days. Recently, improved extraction, cleaning, and quantification methods have been developed. Hence, better recoveries are expected from recent studies on antibiotics degradation during the anaerobic digestion of manure.

**Table 4:** Persistence of major classes of veterinary antibiotics in manure (adapted from Boxall *et al.* [3]).

Chemical group	Half-life (d)	Persistence class
Aminoglycosides	30	Moderately persistent
β-lactams	5	Slightly persistent
Macrolides	<2 to 21	Impersistent to slightly persistent
Quinolones	100	Very persistent
Sulphonamides	<8 to 30	Slightly to moderately persistent
Tetracyclines	100	Very persistent

### 2.2. Biodegradation Level of Antibiotics in Manure Biological Treatment

Bioavailability of antibiotics determines their degradation rate, however, bioavailability depends on the compound's hydrophobicity [54]. Therefore, antibiotic's chemical properties and manure-related matrix characteristics modify the antibiotics' reluctance to biodegradation and play a significant role in antibiotic removal, respectively [50]. Motoyama *et al.* [13] related differences in the measured concentrations of the same antibiotic in different types of manures (swine, cattle, and horses) to the specific adsorption characteristics of the different manures’ matrices. The physicochemical characteristics of various antibiotics correlated with their degradation profiles and support these assumptions [34].

Degradation of antibiotics in compost, soil, manure, and sediments follows the same metabolic mechanisms [55] though differences among different media matrices affect the fractioning of antibiotics between liquid and solid phases. Results of antibiotics’ degradation studies should be considered cautiously depending on how the bioassay has been conducted, the extraction recovery efficiency, and the resolution of the quantification protocol. Studies reporting on degradation of antibiotics as sole substrate using a standardized bacterial consortium in closed bottles incubated in the dark at 20 °C and assessing the oxygen consumed on theoretical oxygen demand (ThOD) [56] provide a limited information. More reliable results should be obtained from studies conducted with antibiotic-containing manure simulating real situations using mixed anaerobic cultures and monitored by gas production, COD removal, and VFAs consumption. Table 5 presents a summary of antibiotics degradation in livestock manure biological treatment. Notice that the removal of oxytetracycline varied from as low as 55%–70% (soil) to 55%–75% (anaerobic digestion) to 85%–99% (composting). Except for the 99% removal during composting, all other removals efficiencies have been achieved using the same initial oxytetracycline concentration (20 mg·L^−1^).

Careful examination of Table 5 reveals several trends. The high removals during composting are likely due to the effects of the additional aerobic bioactivity compared to anaerobic digestion alone. Although both soil and composting share the same aerobic-anoxic conditions composting showed higher removals likely because of the presence of good inoculum compared to soil condition. No sound conclusion could be drawn regarding the effect of the biological action temperature on antibiotic removal. For example, mesophilic and thermophilic anaerobic digestion operation showed higher removals of chlortetracycline than psychrophilic operation, however, for monensin both psychrophilic and mesophilic showed low removals compared to thermophilic. Interestingly, oxytetracycline was removed in soil by almost the same efficiency at 5 °C and 15 °C, while at 25 °C a 20% increase in the removal efficiency was observed.

Although the degradation half-lives were reported for antibiotics in stored solids or in soil where there is a “passive” biodegradation, these values do not reflect degradation rates in the presence of an active biomass such as in waste treatment processes. Aerobic and anaerobic waste treatment processes have shown their efficiency to remove many xenobiotics and pharmaceuticals from effluents. Some studies have evaluated the biodegradability, mostly in aerobic conditions, of various antibiotics used for human health and animal production. Al-Ahmad *et al.* [57] have shown, when testing the biodegradability of cefotiam, ciprofloxacin, meropenem, penicillin G, and sulfamethoxazole using closed bottle test [56], that only pencillin G was biodegradable to some degree (27%), prolonging the test from 28 to 40 days increased the removal to 35%. Using the same test to evaluate the biodegradability of ciproflaxin, ofloxacin and metronidazole, Kümmerer *et al.* [58] observed no biodegradation of those antibiotics, without loss of their genotoxicity.

Wang *et al.* [59] found that degradation kinetics of sulfadimethoxine was affected by its initial concentration because microorganisms are inhibited at high antibiotic concentrations; this result could presumably be extrapolated to any antimicrobials degradation kinetic. Shi *et al.* [60] found that tetracycline and sulfamethoxydiazine initial concentrations of up to 50 mg·L^−1^ decreased by 50% within 12 h of continuous anaerobic digestion (OLR 1.88 kg COD m^−3^·d^−1^) and only traces of antibiotics were detected after 2–3 days. These researchers did not provide evidence whether the reduction of the antibiotics concentration was due to sorption or biodegradation. Loke *et al.* [61] found a half-life value lower than 2 days for tylosin A in manure spiked with 25 mg·L^−1^ during anaerobic digestion of swine waste at 20 °C. Moreover, in aerobic conditions the disappearance rates of tylosin A increased with increasing concentrations of solids, but it was not clear if removal was due to bacterial or abiotic degradation, or that sorption on manure particles was responsible for low aqueous antibiotic concentrations. Loke *et al.* [61] did not observe instant sorption of tylosin on manure particles, with 102% to 108% recoveries during method validation. However, recovery efficiencies were not assessed for long term contact between the antibiotic and manure particles. Hence, it is plausible that the more concentrated solids adsorb more of the antibiotic over time and less is recovered, which gives the impression that the half-life is shorter in this condition.

**Table 5:** Biodegradation of antibiotics in manure.

Treatment	Antibiotic	Concentration	Observed reduction	Reference
**I. Anaerobic digestion**				
Anaerobic digestion of swine manure 21 days	Chlortetracycline	6.5 mg·L^−1^	7% (22 °C)	[62]
		8.3 mg·L^−1^	80% (38 °C)	
		5.9 mg·L^−1^	98% (55 °C)	
Anaerobic digestion of cattle manure (28 days)	Monensin	0.74 mg·L^−1 ^	3% (22 °C)	[62]
		0.36 mg·L^−1^	8% (38 °C)	
		0.30 mg·L^−1^	27% (55 °C)	
Batch anaerobic digestion	Oxytetracycline	20 mg·L^−1^	55%–73% at 37 °C	[63]
Anaerobic sequence batch reactor (ASBR)	Tylosin A	1.6 mg·kg^−1^	Degraded to <detection limit.	[1]
		5.8 mg·kg^−1^	Decreased to 0.01 mg·L^−1^ in 48 h	
Swine manure from lagoons	Tylosin	0–400 mg·kg^−1^	95%–75%	[64]
**II. Composting**				
Composting (22–35 days)	Chlortetracycline	1.5 mg·kg^−1^	99%	[65]
	Monensin	11.9 mg·kg^−1^	54%	[65]
	Tylosin	3.7 mg·kg^−1^	54%	
	Sulfamethazine	10.8 mg·kg^−1^	–76%	[65]
Composting beef manure (35 days) abiotic removal	Oxytetracycline	115 μg·g^−1^ DM	99% (laboratory)25% (22 °C)	[51]
Composting	Oxytetracycline	20 mg·L^−1^	85%	[13]
	Tetracycline		92%	
	Chlortetracycline		90% (all removals	
	Levofloxacine		81% at 38 °C)	
	Ciprofloxacine		100%	
	Erythromycin		67%	
	Sulfamonomethoxine		79%	
	Sulfamethoxazole		95%	
	Trimethoprim		86%	
	Carbamazepine		37%	
**III. Manure amended soil**				
Soil	Tetracycline	5–300 µg·kg^−1^	0%0%	[66]
	Chlortetracycline	4.7 µg·kg^−1^	0%0%	
	Sulphanilamide	0.25–1.0 mg·L^−^^1^	0%	[67]
	Tylosin	5.6 µg·L^−^^1^	0%	[68]
	Erythromecin	5.6 µg·L^−^^1^	25%	[68]
Storage	Sulfadiazine	156 mg·L^−1^	0% (10 °C and 20 °C)	[46]
	Difloxacin	17.6 mg·L^−1^	7% (10 °C and 20 °C)	

The effects of individual and mixtures of antimicrobials on manure biological treatment depend on inhibition and resistance mechanisms, the manure matrix, the composition of the microbial community, biotic and abiotic degradation of antimicrobials, and sorption of antimicrobials [69].

#### 2.2.1. Tetracyclines

Abiotic mechanisms were responsible for a removal of 98% of CTC (initial concentration (C_i_) = 113 µg·g^−1^) during 30 days of beef manure composting (TS = 30%) [9]. However, there was an increased loss of extractable CTC residues with increased time, probably due to sorption to organic matter, rendering its quantification difficult. Approximately 60% removal of OTC (C_i_ = 9.8 mg·L^−1^) was achieved in 64 days by anaerobic digestion at 35 °C (TS 4.0% to 4.7%) yielding a calculated half-life of 56 days for OTC [30,48]. Also, approximately 75% removal of buffer extractable CTC (C_i_ = 5.9 mg·L^−1^) was achieved in 33 days by anaerobic digestion at 35 °C yielding a calculated value half-life of about 18 days. However, these removals during anaerobic digestion cannot be directly related to biological activity or abiotic mechanisms since no sorption analysis was performed. Anaerobic digestion decreased concentrations of OTC from 13.5, 56.9 and 95.0 mg·L^−1^ to 5.7, 26.6 and 30.7 mg·L^−1^ in 21 days, respectively, while CTC was decreased from 9.8, 46.1 and 74.0 mg·L^−1^ to 0.9, 4.0 and 7.5 mg·L^−1^, respectively [70]. CTC was transformed and epimerized at faster rates than that for OTC. CTC decreased in the solid fraction at a slower rate than that observed in the aqueous phase likely because water-extractable antibiotics are most “available” for degradation by microorganisms and that 100% of CTC concentration has been found water-extractable [65]. Finally, the degradation product and epimer of CTC, 4-epi-chlortetracycline (ECTC), was completely removed at high rate [70]. Arikan *et al.* [48] and [30] reported a significant removal of the parent compounds of CTC and OTC during the first 10 days of incubation, then, OTC was degraded in 60–70 days whereas CTC was removed at a slower rate. These finding agrees with the half-life of OTC (22–27 days) determined during batch anaerobic digestion of manure [63].

The adsorption of OTC and CTC was limited by the available superficial area of the inoculum and pig manure [70]. Furthermore, OTC and CTC form strong complexes with divalent cations, which are abundant in pig manure, adsorb onto proteins, particles and organic matter [71]. At 35 °C and pH 7, 40% and 60% of OTC and CTC, respectively, were removed in the first hour. 4-epi-oxytetracycline (EOTC), an epimer of OTC and ECTC degraded quickly. After 7 days, 6% of the initial amount of OTC remained in the assay [70].

Álvarez *et al.* [70] determined also the first-order degradation constants for OTC (0.045 to 0.058 d^−1^) and CTC (0.169 to 0.216 d^−1^) while Arikan *et al.* [30] reported lower first-order degradation constants (0.012 and 0.039 d^−1^ for OTC and CTC, respectively). This inconsistency might have been caused by the higher organic matter content in the assays (50 g·L^−1^ of cattle manure), which could have increased the stability of both compounds due to their strong adsorption onto the solid fraction [70].

The half-life of oxytracycline in manure was 30 days and it was detectable (820 ug·kg^−1^) after 5 months of maturation [35]. Søeborg *et al.* [72] suggested that some portion of chlortetracycline degradation during composting may be due to abiotic processes.

#### 2.2.2. Tylosin

De Liguoro *et al.* [35] found that tylosin degraded rapidly and it was undetectable in manure after 45 days; no trace (>10 ug·L^−1^) of the compound was detected in soil or surrounding water. Chelliapan *et al.* [64] reported that 95% tylosin reduction with a COD reduction of 93% were achieved in an up-flow anaerobic stage reactor (UASR) treating pharmaceutical wastewater (contains tylosin 0 to 400 mg·L^−1^) at a HRT of 4 d and OLR of 1.86 kg COD m^−3^·d^−1^. However, at concentrations of 600 and 800 mg·L^−1^ the COD reduction was 85% and the tylosin removal was 75% [64]. They concluded that tylosin concentrations ≤ 400 mg·L^−1^ had a minimal effect on reactor performance. Methanogens were active in the reactor even at 800 mg·L^−1^ tylosin which did not affect the CH_4_ yield. Similar findings that such as high concentrations of tylosin are unlikely to create problems in the treatment of wastewater by anaerobic digestion have been reported in other studies [73,74].

The tylosin A half-life of (2.5 h) in high-rate anaerobic digester is shorter than its half-lives (2–8 days) in soils or passively stored manure [1,41,61,75]. Tylosin was degraded in soil columns (half-life 3.3–8.1 days) [41]. Kolz *et al.* [27] found that 90% of tylosin A in anaerobic sludge was sorbed and degraded (abiotic or biotic) within 5 days in anaerobic digestion. Angenent *et al.* [1] concluded that tylosin was removed by degradation rather than sorption in anaerobic batch experiment and ASBR; with dehydroxy-tylonolide as a by-product. Such conclusion could be explained by the fact that water-extractable antibiotics are most “available” for degradation by microorganisms given that 85% of the total-extractable concentration of tylosin was water-extractable [65].

Chelliapan *et al.* [76] reported tylosin (initial concentration 10–220 mg·L^−1^) removal of 70–88% in upflow anaerobic stage reactor operating at OLR 1.86 kg COD m^−3^·d^−1^ with a COD removal of 70%–75%. At higher OLR (2.84–3.73 kg COD m^−3^·d^−1^), tylosin removal increased and was stable between 93%–99% despite that the COD removal declined to about 45%. Obviously, there is no agreement on which mechanism is responsible for the removal of tylosin in anaerobic digestion.

#### 2.2.3. Other Antibiotics

Sulfamethazole was utilized as carbon and nitrogen source by the microorganisms in absence of those nutrients, but remained intact in the presence of acetate and ammonium [77]. Antibiotics like ciprofloxacin, ofloxacin, and virginiamycin degrade very slowly and may persist in soil in its original form up to 30–80 days while bambermycin and erythromycin completely degrade in a period of one month at temperatures ranging from 20–30 °C [21].

Carballa *et al.* [78] found that mesophilic anaerobic digestion (STR of 30 days) degraded 99 and 94% of sulfamethoxazole, and roxithromycin, respectively. Water-extractable antibiotics are most “available” for degradation by microorganisms. The percentage of initial water-extractable antibiotic concentration out of the total-extractable was 40% for monensinand 85% for sulfamethazine [65].

Kim *et al.* [79] observed decrease in tetracyclines, sulfamethazine, and tylosin concentrations from 20 mg·kg^−1^ to less than 0.8, 0.2, and 1.0 mg·kg^−1^, respectively, during composting of pig manure with saw dust. Presence of saw dust correlated with the decline in tetracyclines and sulfamethazine concentrations, but not with tylosin. Again, it is debatable to compare results from different studies because of the utilization of various antibiotic quantification techniques having different reliabilities and precision. Moreover, most of these studies did not discuss the possibility that antibiotics would be adsorbed on particles and thus not quantified, biasing the degradation rates obtained.

### 2.4. Metabolites

Fedler and Day [80] suggested that the antibiotics themselves may not inhibit bacteria but their metabolites produced in the gastrointestinal tract of the animal may. 4-Epi-oxytetracycline (EOTC), a-apo-oxytetracycline (a-Apo-OTC) and b-apo-oxytetracycline (b-Apo-OTC) are degradation products of oxytetracycline (OTC) [48] whereas 4-epi-chlortetracycline (ECTC) is a degradation product and epimer of chlortetracycline (CTC). These metabolites are similar to their parent in creating complexes with metal ions, humic acids, proteins, particles and organic matter in the manure matrix [71] thus they are strongly adsorbed in manure. Unfortunately, almost all of the studies on antibiotics in manure focused on the parent compounds except several studies on OTC and CTC where antibiotic degradation progenies were monitored. It has been concluded that antibiotic metabolites produced in the gastrointestinal tract of the animal may inhibit bacterial activity more than the original molecule [73]. On the contrary, Halling-Sørensen *et al.* [81] found that the degradation products of OTC have less biological activity on sludge and soil bacteria than OTC. These authors also found a similar trend of biotransformation between the parent and the intermediate compounds (EOTC and ECTC), as well as the removal of these intermediates [70].

## 3. Required Future Research

Developing standard protocols to assess the impact, degradation, and fate of various antibiotics and their metabolites during anaerobic digestion is essential to enable a reasonable comparison among results generated from different studies. Assessing the effect of culture matrices, solid content, and nature of manure organic fraction on the degradation dynamic of various antibiotic during anaerobic digestion is required with a focus on kinetic and metabolic modeling and simulation of inhibition, recovery, and adaptation mechanisms. Particularly, better understanding and prediction of the contribution of abiotic (physical and chemical) *versus* biotic degradation mechanisms of the different antibiotic classes is required. Fractioning of manure-laden antibiotics into liquid and solid phases and its effects on their anaerobic degradation needs to be understood and quantified under various manure classes, antibiotics types, reactor design and operation. For design purposes, kinetic data is required for the degradation of the antibiotic parent compounds and their metabolites in different anaerobic reactor designs and operation. Effects of process staging and modification need to be explored to avoid operational problems due to the effects of high antibiotics’ concentrations on the anaerobic digestion. Potential of psychrophilic anaerobic digestion of livestock manure to eliminate antibiotics and antibiotic resistant bacteria has not been investigated yet.

## 4. Conclusions

Most antibiotics form complexes with metals and soluble organics in manure and remain stable during storage, however; anaerobic digestion can degrade them to various extents depending on the concentration and class of antibiotic, operation condition, and type of culture.

Antibiotic’s chemical properties and manure-related matrix characteristics interact to modify their reluctance to biodegradation and play a significant role in antibiotics' removal. The physicochemical characteristics of various antibiotics correlate with their degradation profile. Antibiotics' degradation during anaerobic digestion depends on their water-extractability which affects their bioavailability to microorganisms. Therefore, fractioning of antibiotics into liquid and solid phases of manure, its effects on their anaerobic degradation, and contribution of abiotic (physical and chemical) *versus* biotic degradation mechanisms need to be determined and quantified for various manures, antibiotics types, reactor designs and operation conditions. Different types of biological processes affect antibiotic persistence differently; composting > anaerobic digestion > soil.

Antibiotics and their metabolites are strongly adsorbed in manure because of chemical combination with metals and organics. More research is required to evaluate kinetics and fate of antibiotic degradation progenies. It is strongly suggested that standard analytical protocols be developed for the detection, extraction, and quantification antibiotics from manure. Such standard methods will enable sound comparison of the results generated from different studies and making better conclusion regarding the impact, degradation, and fate of various antibiotics and their metabolites during anaerobic digestion. Assessing the effect of culture matrices, solid content, and nature of manure organic fraction on the degradation kinetics of various antibiotics during anaerobic digestion is required with a focus on kinetic, metabolic modeling, simulation of inhibition, recovery, and adaptation mechanisms. Further investigations are required to assess the degradation of antibiotics during psychrophilic anaerobic digestion.
